# Towards Better Culturally Tailored Cardiometabolic Prevention Among the South-Asian Surinamese in the Netherlands

**DOI:** 10.3389/ijph.2023.1606380

**Published:** 2023-11-28

**Authors:** Helene R. Voogdt-Pruis, Lieke van den Brekel, Lian Wispelweij, Laxmie Jawalapershad, Soerin Narain, Ilonca C. H. Vaartjes, Diederick E. Grobbee, Kerstin Klipstein-Grobusch

**Affiliations:** ^1^ Department of Global Public Health and Bioethics, Julius Center for Health Sciences and Primary Care, University Medical Center Utrecht, Utrecht University, Utrecht, Netherlands; ^2^ Stichting Ester, Den Haag, Netherlands; ^3^ Stichting Vobis, Den Haag, Netherlands; ^4^ Division of Epidemiology and Biostatistics, School of Public Health, Faculty of Health Sciences, University of the Witwatersrand, Johannesburg, South Africa

**Keywords:** prevention and control, healthy lifestyle, cultural adaptation, social network, South-Asian people

## Abstract

**Objectives:** To gain insight in the motives and determinants for the uptake of healthy lifestyles by South-Asian Surinamese people to identify needs and engagement strategies for healthy lifestyle support.

**Methods:** We used a mixed-method design: first, focus groups with South-Asian Surinamese women; second, a questionnaire directed at their social network, and third, interviews with health professionals. Qualitative content analysis, basic statistical analyses and triangulation of data were applied.

**Results:** Sixty people participated (*n* = 30 women, *n* = 20 social network, *n* = 10 professionals). Respondent groups reported similar motives and determinants for healthy lifestyles. In general, cardiometabolic prevention was in line with the perspectives and needs of South-Asian Surinamese. However, there seems to be a mismatch too: South-Asian Surinamese people missed a culturally sensitive approach, whereas professionals experienced difficulty with patient adherence. Incremental changes to current lifestyles; including the social network, and an encouraging approach seem to be key points for improvement of professional cardiometabolic prevention.

**Conclusion:** Some key points for better culturally tailoring of preventive interventions would meet the needs and preferences of the South-Asian Surinamese living in the Netherlands.

## Introduction

People of South Asian ancestry, such as the South-Asian Surinamese living in the Netherlands, have a disproportionately higher burden of cardiometabolic diseases when compared to other population groups. They have a higher cardiometabolic disease risk and a higher prevalence of diabetes and obesity. A complex interplay of factors including lifestyle habits, social factors and broader environmental factors contribute to this higher burden [1–5]. South-Asian Surinamese make up one of the largest minority groups in the Netherlands [5, 6]; they immigrated to the Netherlands after Suriname, a former Dutch colony in South America, gained independence in 1975. Their ancestors were born in India and were recruited to work as indentured labourers in Suriname after the abolition of slavery [7]. Most of the South-Asian Surinamese live in one of the four major Dutch cities, including The Hague [5, 6], often with lower socioeconomic positions with limited access to resources in society such as finances, education, and social network, and living in less healthy environments [6, 8, 9]. As cardiometabolic health is determined by individual factors as well as social, economic, and environmental conditions, preventive interventions are manifold, yet still face the challenge of achieving a sustained change to healthy lifestyles [1, 8–10]. Efforts to develop culturally adapted lifestyle intervention programmes for minority groups seem to be promising. By cultural tailoring of interventions, the design of preventive programmes and materials are adapted to the cultural needs and preferences at the population level. Culturally adaption of preventive interventions could be achieved by taking into account cultural relevant values of family and social network, the current diet and physical activity practices, the culturally relevant celebrations and gatherings and the living conditions or constraints such as weather and restricted budgets [11]. However, the effectiveness of these interventions in daily practice has not been conclusively shown and questions have been raised as to whether the interventions really do meet the needs and preferences of minority groups and whether these are truly imbedded in the daily practice of health professionals [1, 12, 13]. A co-design with the target group in developing or adapting preventive interventions—such as working with key persons from within the community—and the support of the local community (e.g., to create appropriate environments that could facilitate the participation of minority communities) seems to be important for achieving effective prevention programmes [12–15]. We conducted a study in The Haque to explore the perspectives on healthy lifestyles and lifestyle support and what was needed in that regard among the South-Asian Surinamese and how the social network (family, friends and neighbours) and professional public services are facilitating the South-Asian Surinamese to achieve healthy lifestyles. Most of the South-Asian Surinamese live in the deprived neighbourhoods of The Haque—with lower life expectancy and lower income compared to other neighbourhoods [6].

## Methods

### Study Design

This qualitative study used a sequential explorative design [16]. During May and June 2022, four focus group sessions were held to explore the way healthy lifestyles were perceived among women with South-Asian Surinamese backgrounds. In addition, in July 2022, the social and community networks of these women were approached for a short questionnaire. Finally, in August 2022, professionals working in the local health services of The Haque were invited for an online interview.

### Research Team

The researchers conducted the study in cooperation with two key persons of the South-Asian Surinamese ethnic group (LJ, SN). These key persons were recruited via Pharos, a Dutch centre of expertise on health disparities. Both key persons were involved as volunteers in the South-Asian Surinamese community in The Hague through the Vobis Foundation, a non-profit foundation that supports marginalised groups towards social and economic independence and promotes social cohesion. The community centre of the Vobis Foundation is situated in the neighbourhood “Moerwijk,” in The Haque. Therefore, we conducted our study in The Haque.

### Data Collection

The setup of the study was based on Dahlgren and Whitehead’s “Determinants of Health” model [10], in which health is determined by individual factors, by determinants related to the social and community networks, and also by factors related to healthcare services.

Step 1. Four focus group sessions with women: Focus group sessions were used to gain a better understanding of the needs and preferences towards healthy lifestyle and preventive interventions and the values and social norms within the South-Asian Surinamese population living in the Netherlands [17]. Women aged 18 years or older with a Surinamese-Hindustani background living in The Hague, were eligible for participation. The key persons recruited eligible women through their social and personal network. Although not a strict criterion, recruitment of participants with a self-reported history of cardiovascular disease (CVD), type II diabetes mellitus, obesity, metabolic syndrome, rheumatoid arthritis, hypertension or hypercholesterolemia was prioritised. The key persons of our research team (SN, LJ) recommended only inviting women to the focus groups as women are traditionally involved in the tasks of grocery shopping and cooking for the household, influencing the dietary habits of the entire household. This would encourage more openness in the discussions [18]. Of course, women in the focus groups shared about lifestyle habits of relatives and husbands too. The focus group sessions were held at the local community centre, in a setting where most of the participants usually meet, to provide a safe and familiar environment where they would feel comfortable to speak freely. The key persons had experience of leading discussion meetings. They were informed about the inclusion criteria for participation and they received an information letter about the focus group study. Multiple meetings between the key persons and researchers took place to educate the key persons on the research topics and on leading a focus group session. The key persons set the date for the focus group discussions in collaboration with the women and researchers. All focus group sessions were moderated by one of the key persons (SN, LJ). A researcher from the research team assisted as the second moderator (LW, LB, HV) and ensured that all themes were covered during the sessions. If necessary, the second moderator asked the participants additional questions or provided explanations of the study or themes. The focus group sessions were held in Dutch and backed up by the mother tongue, Sarnami Hindustani: if participants could not get their answers across in Dutch, they were encouraged to express themselves in their own language and the moderator would translate it for the assisting researcher. Informed consent and permission for audio recordings of the focus group discussions were received from both the participants and the key persons and stored on audio tapes. Focus group participants received a voucher and the key persons received financial remuneration for their contribution to the study. Each focus group meeting consisted of three different rounds of discussions with the themes of 1) health and healthy living; 2) the influence of social and professional networks on health or healthy living, and 3) visiting green spaces. For the opening questions on healthy lifestyles, we used one slide of pictures about food, drinks, exercise and daily life. The questions aimed to elicit motives for healthy lifestyles, perceived barriers and facilitators, preferred formats or methods of support either by the social network or by health or healthcare professionals. The third theme, “visiting green spaces,” will be addressed in another manuscript by the research team.

Step 2. The informal social network, including family, friends, and neighbours: Women who participated in the focus groups received a short questionnaire on paper at the end of the meeting. They were asked to give the questionnaire to a family member outside their own household or a friend or a neighbour. We decided to use a short questionnaire for this group in order to verify the answers on the impact of social network as expressed by in the focus group sessions and to ask some additional questions. The short questionnaire addressed three topics about healthy lifestyles, using mainly closed questions: who influenced your health and healthy living (e.g., diet, drinking, exercise, fitness, use of medication), how did those people influence your health and healthy living, and what characterised that influence as positive or negative? In that way, we also gained an understanding of the perspectives and needs relating to healthy lifestyles and lifestyle support of the social informal network of the participants in the focus groups. We asked the women to get the questionnaires filled in within one week. Respondents to the questionnaire remained anonymous. The completed questionnaire could be submitted through WhatsApp to one of the researchers (HV).

Step 3. The formal public health network including GPs, practice assistants, community coaches, physiotherapists and dieticians: In several neighbourhoods in The Hague that are popular with the South-Asian Surinamese community, health professionals were approached about participating in a semi-structured interview lasting about 15 min (via MS Teams). Contact details of health professionals were obtained through the key persons and by accessing websites of health professionals in the neighbourhood. We approached all professionals by mail and/or telephone to obtain 10–15 interviews considered to reach data-saturation. We preferred the interview method as this allowed us to delve deeper into answers given. In that way, we tried to gain an understanding of the perspectives and experiences on lifestyle support from professionals for the South-Asian Surinamese community, and which barriers and facilitators they experience in daily practice. The interview with the professionals covered two main topics concerning the alignment of preventive measures with the needs of the South-Asian Surinamese (How do you boost the health of ethnic groups, and of the South-Asian Surinamese in particular? How much does this meet their needs?).

### Data Analyses

Step 1. Focus group sessions with South-Asian Surinamese women: The audio tapes of the focus group sessions were transcribed verbatim. Qualitative content analysis was applied to the transcripts using NVIVO Version 12.7.0. Three researchers (HV, LW, LB) read all the transcripts independently and assigned codes according to the main themes. After coding the first transcript, a first draft of the codebook was developed in a discussion meeting about the codes and categorisation of codes. This first draft codebook was used for analysing all four transcripts again, one transcript by two of the researchers. In the final step of the analysis, the three researchers discussed the differences in codes again and re-categorised codes (and underlining quotes) according to the final version of the codebook. The results were shared and cross-checked with the whole research team and with the key persons.

Step 2. The informal social network, including family, friends and neighbours: All answers to the open and closed questions of the written questionnaires were stored in an Excel data file and frequencies were retrieved with SPSS 28.0 after data cleaning.

Step 3. The formal public health network including GPs, practice assistants, community coaches, physiotherapists and dieticians: Transcripts of the interviews were analysed by the two researchers (HV, LW) and a summary of the answers was formulated jointly. Once data saturation was reached, data collection was completed. The focus group discussions results were shared and discussed with the respondents.

Final step: By triangulation of the data of the three preceding steps, we obtained more reliable answers to our research questions. In our study, each step included a different target group and research method, with different key sources of potential bias that are unrelated to each other. By comparing and analysing the results of different approaches and finding similar phenomena, the confidence in the findings and conclusions are strengthened. In that way, triangulation of data provided us a better idea of the needs and capabilities for engaging with local health promotion activities. This could guide future development of tailored preventive interventions [17].

## Results

### Participants

In total, 60 people participated in the study: 30 South-Asian Surinamese people joined the focus group sessions (Step 1), 20 people from their informal networks filled out the questionnaire (Step 2) and 10 health professionals participated in the interviews (Step 3). Participants in the focus group study (Step 1) had a mean age of 61 years (SD = 12.6) and were mainly living alone (70%), in disadvantaged neighbourhoods (77%) (Table 1). A self-perceived elevated risk for cardiometabolic diseases was found among 60% of the participants and the majority (94%) were born in Suriname. Respondents to the informal network questionnaire (Step 2) had a mean age of 39 years (SD = 16.7), were mainly women (70%, *n* = 20) and 20% had a self-reported low educational level (*n* = 20). Ten health professionals with a different background participated in the third round: interviews were held with three community workers, two general practitioners, two dieticians, one nurse, one physiotherapist and one public health worker. Nearly all had at least 5 years of relevant work experience (90%).

**TABLE 1:** Characteristics of the participants: focus groups (*n* = 30) and survey in social network (*n* = 20) (Netherlands, May and June 2022).

Four focus groups	*n* = 30
Age in years (mean/SD)	60.6/12.6
Self-reported increased risk of cardiometabolic diseases (%)^a^	60
Born in Suriname (%)^b^	94
Living alone/single household (%)^b^	∼70
Disadvantaged neighbourhood (%)^c^	77
Survey in social network (response rate in %)	*n* = 20 (66.7)
Female (%)	70
Age in years (mean/SD)	38.8/16.7
Self-reported low level of education (%)^d^	20
Self-reported medium level of education (%)	60
Interviews with health professionals	*n* = 10
Type of professional (*n*)	
Community worker	3
General practitioner	2
Dietician	2
Practice nurse	1
Physiotherapist	1
Years of experience—portion > 5 years (%)	90

^a^ Defined as self-reported history of a cardiovascular event, type II diabetes mellitus, obesity, metabolic syndrome, rheumatoid arthritis, hypertension or hypercholesterolemia.

^b^ Reported by the key persons.

^c^ Determined by a higher % of households with income below the minimum compared to the average for the respective cities.

^d^ Self-reported educational level. Most respondents recorded the type of education. Low level means primary school only.

### Factors Influencing the Uptake of Healthy Lifestyle by South-Asian Surinamese People

In response to the question “What do you do to lead a healthy life?” the South-Asian Surinamese people mentioned the following aspects in random order: 1) healthy diet; 2) exercise; 3) relaxation; 4) sleep; 5) social encounters; 6) professional care and 7) - if applicable - proper use of medication (Table 2). Seven main facilitating or hindering factors for a healthy lifestyle were reported: 1) cultural customs; 2) physical health; 3) motivation; 4) time; 5) mental resilience; 6) social network and 7) language barriers in professional lifestyle support (Table 2). In addition, the South-Asian Surinamese described seven main motives for a healthy lifestyle: 1) losing weight; 2) feeling fit; 3) being able to relax; 4) living longer; 5) preventing diseases (including ones that run in the family); 6) being there for children and grandchildren and not being a burden on them and 7) being less incapacitated by illness (Table 2).

**TABLE 2:** A healthy lifestyle, according to South-Asian Surinamese women (Netherlands, May and June 2022).

Themes	Example quotes from participants to the 4 focus groups
A. What do you do to lead a healthy life?	
Healthy diet: Eat more/less of certain products; Eat less or go on diets; Do my own cooking	*I hardly eat any rice and I do avoid roti, much as I love it. [FG3] We use a lot of spices in the food and I have to tone that down a bit. [FG3] Steam things, with less fats and oils. [FG1] I use red fermented rice along with brown rice and that brought my blood pressure down. [FG3]*
Exercise: Walk or cycle; Exercises in the home; Play sports	*I can walk for hours—alone, because I don’t reach my target counts if I go with others. [FG3] I did the cleaning today, so that’s exercise as well. [FG1] I love dancing and I’ll keep dancing until I can’t do it anymore. [FG1]*
Relaxation: Creative activities; Spirituality; Yoga; Meditation	*At home, I meditate—it gives me inner peace. [FG1] Living healthy is also about letting go of the stress. [FG2] Nothing interested me anymore and I was really lonely. Then I found God and there were lovely people in the church who really talk to you. [FG4]*
Sleep	*I try to get to sleep in good time. I don’t stare at a screen after nine and don’t mess around with my mobile. [FG1]*
Social encounters	*The healthiest people are in the blue zones It’s because of the social contacts. [FG2] I go for a walk every Sunday with my neighbour [FG4]*
Professional care	*I have three injections a day and I’m monitored closely. [FG3] We need a dietician who doesn’t just keep telling us we’re not allowed rice. What are we supposed to swap it for? We eat very differently [FG4]*
Medication	*Exercising and watching my diet meant I was able to stop taking my pills. [FG1]*
B. What factors play a role?	
Cultural customs	*It’s difficult to say “no”. That’s not part of our culture [FG4] I’ve got to have just a bit of ordinary rice every day [FG1]*
Physical health	*That’s part of the luxury situation we’re in here. We don’t get much exercise and we eat a lot. Not doing my own cooking, eating out too much [FG2]*
Motivation	*I eat everything: samosas, curries—tasty stuff. I want to eat healthily but if the food isn’t very nice then, I won’t eat it [FG4] Sure, you can diet but then you’re taking all the fun out of life [S2]*
Time	*I find it easy to eat healthily—it’s become my natural pattern [S4] I stick to the structure, otherwise I fall back into my old habits too easily [S1]*
Mental resilience	*If I’m unhappy, I don’t want food. When I’m feeling good, I eat a lot [S1]*
Social network	*I’d really like to learn how you keep away from all the rice [S1]*
Language barrier	*We don’t always understand the Dutch phrasing used by the professionals [S4]*
C. Why live a healthy life?	
To lose weight	*Then I won’t get too fat. [S2]*
To feel fit	*At first, I felt tired when I’d been walking but now that I’ve lost weight, the only thing that bothers me a bit is my knee. My cholesterol is fine. [S1]*
To relax	*If you go out for a walk, it clears your mind. [S2]*
To live longer	*I want to live to a ripe old age. [S2]*
To prevent diseases (including ones that run in the family)	*If you exercise, there’s less chance of dementia. [S1] My brothers and sisters are diabetic too. I’m careful. [S4]*
To be there for my children and grandchildren and not be a burden on them	*I want to set a good example for my kids because then they’ll copy me. [S2]. For my grandchildren above all, so that I’ll be able to enjoy being with them for longer. [S1] I don’t want my family to have to look after me and I don’t want to be a burden to them. [S4]*
To be less incapacitated by my illness	*I already had that issue, but if I’d gone on the way I’d been going, I might well have been paralyzed by now. [S1]*

### Towards a Better Culturally Tailoring of Prevention for the South-Asian Surinamese

Triangulation of the findings of the focus group sessions with data from the questionnaire among the social network and data from the interviews among local health professionals (Table 3) provided us with several important insights to better facilitate culturally tailoring of preventive intervention for the South-Asian Surinamese. Professional cardiometabolic prevention could be improved by paying more attention to the following aspects: • Search for incremental changes to current lifestyles

**TABLE 3:** Impact of the social network and health professional on healthy lifestyles of South-Asian Surinamese (Netherlands, May and June 2022).

Expressed by participants of the focus group (*n* = 30)			
Whose influence?	Family, friends, dietician, GP, other professionals, neighbours, clubs, influencers	*[Some example quotes]: If you want to eat healthily and your family want to eat something and you don’t, you have to be able to handle that. [FG1] I give healthy tips to my friends [FG1] We need a dietician who doesn’t just keep telling us we’re not allowed rice. What are we supposed to swap it for? [FG4] The coach is too strict. You can’t actually live that way [FG4] I’ve gone walking with others, but they end up sitting down because they’re tired. [FG2] The discussion groups with women—that helps a lot [FG1]*	
How?	Advice		
	Swapping tips		
	Exercising together		
	Eating together		
	Talking about it		
Expressed by the social network (*n* = 20)—Commonest responses			
Whose influence?	other relatives, friends; parents, grandparents; GP, nurse practitioner; children; partner; others	
How?	1. In daily conversations	*[Main response]: Mainly a positive influence. Role models in the family are observed. People want to stay healthy for their children. Unhealthy behaviour around you does have a negative influence*	
	2. Advice on exercise or nutrition		
	3. Eating, exercising together		
	4. Conversation about medication use		
	5. Via media (folders, magazines, TV)		
	6. Adopting a habit in the group		
Expressed by health professionals working in the neighbourhood (*n* = 10)			
How do you boost the health of ethnic groups?	*[Some example quotes]: Population-based apporach, whereby the healthcare focuses on the individual. (GP) No specific approach for a specific target group. Surinamese-Hindustani people are more likely to have diabetes and cardiovascular diseases so diagnostic tests are done sooner. (GP) Change diet based on eating patterns and results of lab tests. Change is harder for older people (Dietician) Tailoring to the individual eating pattern. An open approach. Small-scale targets (Dietician) No different approach for specific ethnic groups. Teach them how to cope with COPD (Nurse) We work with a specific method: “Powerful Basic Care” and the 4D model. (Physiotherapist) Support from professionals in the approach for healthy weight in children (Municipal Health Service) Some of them don’t want anything and you have to accept that. (Neighbourhood coach) Mainly geared to women—play sports separately from men, supervised by a woman. (Neighbourhood coach) Sporting activities. No specific approach. Separate activities for women because they don’t always want to do sports with men or because they want to take a certain festival day into account (neighbourhood coach). Collaborate with key persons, the welfare sector or neighbourhood coaches in the district (GP)*		
How much does this meet the needs of ethnic groups?	*[Some example quotes]: Sometimes difficult to persuade people to come to check-ups or take their medication. That stands out with the Surinamese group. Different picture of what is overweight and what isn’t. (GP) A striking aspect is the failure to follow recommendations: not being able or willing to believe the need for it when the results show they have to inject insulin. I have to keep on at them (Dietician) Mainly explaining different kinds of products. They find it incredibly difficult to use different products. They really don’t like the taste of brown rice. (Dietician) Some are happy with the guidance and others less so, and/or they drop out (Nurse) These don’t always have money for physio or for sports. The elderly don’t really like exercise. (Physiotherapist) There is a lot of unhealthy food on offer in the district and a lack of knowledge about products and the importance of exercise. (Municipal Health Service) They mainly look for contact with other people from the same ethnic group. That applies to sports and exercise too. Muslim women only want to do things with other women. (Neighbourhood coach) Activities are held within a community centre; easy access for the target group. (Neighbourhood coach) Most appreciate the fact that we don’t make a distinction based on background. (Neighbourhood coach)*		

Professional support will be needed that promote changes to diet and physical activity that works with current practices and preferences. The introducing of incremental changes seems to work the best. The following examples by participants were given:

I steam things, and use less fats and oils [FG1].

I’ve got to have just a bit of ordinary rice every day. I’d really like to learn how you keep away from all the rice [FG1].

I use red fermented rice along with brown rice and that brought my blood pressure down [FG3].

I did the cleaning today, so that’s exercise as well [FG1].

I love dancing and I’ll keep dancing until I can’t do it anymore [FG1].

• Embrace the importance of the social network and the community

The social network seems to have an important impact on individuals’ lifestyle. Daily conversations, advice, exchanging tips, exercising and eating together, talking about it and/or role models within the social network: it all generates an influence on health and healthy lifestyles (Table 3). Therefore, a culturally tailored prevention should embrace this aspect of a collective culture and pay extra attention to social influences: How to influence the health choices of relatives and how to deal with the impact of others on own behaviour?

It’s difficult to say “no” to food when served. That’s not part of our culture [FG4].

If you want to eat healthily and your family want to eat something and you don’t, you have to be able to handle that [FG1].

I give healthy tips to my friends [FG1].

The discussion groups with women talking to each other—that helps a lot [FG1].

Unhealthy behaviour around you does have a negative influence [FG3].

• Apply an encouraging and user-centred approach

On the one hand, the South-Asian Surinamese participants perceived that they were not properly understood by health professionals and on the other hand, health professionals expressed the difficulty to get their South-Asian Surinamese clients or patients motivated to live a healthy lifestyle. In addition, as most of the women belonged to the first migrant generation, “language barriers” were considered to be difficult to overcome to engage and receive support from health professionals. Finally, some participants regarded the approach by health professionals too strict instead of encouraging.

[Clients/patients]

We need a dietician who doesn’t just keep telling us we’re not allowed rice. What are we supposed to swap it for? We eat very differently [FG4].

We don’t always understand the Dutch words used by professionals [FG4].

The coach is too strict. You can’t actually live that way—you wouldn’t be allowed to eat anything [FG4].

[Professionals]

The Surinamese group have a different perception of what is overweight and what isn’t (GP).

A striking aspect is the failure to follow recommendations: not being able or willing to believe the need for it when the results show they have to inject insulin. I have to keep on at them (Dietician).

It is sometimes difficult to persuade people to come to check-ups or take their medication (GP).

## Discussion

Although the general focus of prevention is shifting from interventions on lifestyle behaviour towards a system approach targeting all driving forces for health [19, 20], our study still provides key points for improvement of daily preventive intervention programmes addressing the first and second-level factors of the social determinants of health (i.e., individual factors, determinants related to social and community networks, determinants of the public and primary healthcare) [10]. In our multi-stakeholder qualitative study among the South-Asian Surinamese community living in The Hague, we gained insights into the perspectives, needs and abilities for engaging with healthy daily lifestyles and preventive interventions. In general, preventive interventions seem to be in line with the perspectives and needs of South-Asian Surinamese but we did observe a mismatch between supply and demand of lifestyle support too. Improvements can be achieved by better culturally tailoring of preventive interventions.

We found similar motivations, barriers and facilitators for healthy lifestyles and lifestyle support among the South-Asian Surinamese compared to other population groups. Healthy lifestyles comprise a healthy diet, exercise, relaxation, sleep, and social encounters [21–26]. In addition to this, people who receive secondary prevention also regarded “visiting a health professional” and “compliance with medication” as health behaviour. With respect to motives for healthy lifestyles our results are in line with those from other studies: losing weight; feeling fit, being able to relax; living longer; preventing diseases; being there for children and grandchildren, being less incapacitated by illness [21–26]. The lack of physical possibilities, motivation, time, or mental resilience previously cited by other studies [21–26] were also mentioned as barriers or facilitators for a healthy lifestyle by our South-Asian Surinamese group. In addition, use of the mother tongue, an interpreter or visuals for communication of preventive measures are in particular important for ethnic minorities. And health professionals should also be open to an individual’s own views. Finance—as a factor for healthy lifestyles and lifestyle support - was not mentioned by our South-Asian Surinamese participants.

We observed a mismatch between supply and demand of lifestyle support: the South-Asian Surinamese expressed other needs for lifestyle support towards exercise and diet [12]. For example, traditional physical exercise programmes focus on fitness, sports class, cycling and walking, either alone or with a group. Nevertheless, South-Asian Surinamese women prefer other ways of exercise, such as dancing together; a recent study underlined the benefits of a dancing intervention in improving physical activity [27]. As South Asians tend to be less physically active than other ethnic groups, recommendations on physical exercise should take account of these kinds of preferences [18] and other views on exercise in the social network. Concerning diet, there is a need for support for healthy diet, food choices and cooking within the South-Asian Surinamese cooking and eating habits [28]. A recent study showed benefits for cardiometabolic health of a dietician-led educational short course on healthy South Asian cooking and meal choices for the family (interpreting food labels, types of oil used in cooking, healthy vegetarian food choices) [29]. Still, gender roles and other influences from the social network might be a barrier for women to modify the household’s diet as others in the household—mostly the male or the elderly—exert influence over dietary decisions [18]. For both support on exercise and diet, attention should be paid to the impact others have on changing and/or maintaining a healthy lifestyle.

Studies have shown that culturally tailored lifestyle intervention programmes are promising in improving cardiometabolic health for South Asians and for self-management, but the urgency to co-design interventions with users seems to be the challenge for daily practice [11, 21–26, 29]. Another challenge is to achieve behavioural change in the long term by creating supportive social networks around individuals within the community. Cultural customs and social networks are unique for different populations. As such, understanding how health behaviours are rooted in an individual’s cultural background and how they respond to social pressures can better equip health professionals for a culturally sensitive approach in cardiometabolic prevention [30–33]. Experts suggested that health professionals should use “mini-ethnography” to understand how identity and perspectives on healthy lifestyles are linked to cardiometabolic health, and how moral and social values impact an individual’s lifestyle [34, 35]. In addition, materials and professional support—including tools for self-monitoring, risk communication and social support—should be developed in co-creation with the respective population group for subsequent implementation [36–38]. Finally, public authorities should support local collaborative prevention networks involving health professionals and community stakeholders that are needed to develop healthier habits [11, 39]. Taking into account that in general health professionals are highly respected and influential in South Asian cultures, prevention by professionals towards South-Asian Surinamese and other ethnic minorities might benefit from a more and better implemented culturally sensitive and social network approach.

### Strengths and Limitations

A strength of the study was the collection and triangulation of three methods: first, focus groups with South Asian-Surinamese women; second, a short questionnaire directed at the social network of the participating women, and third, interviews with professionals working in prevention programs with South Asian-Surinamese. This triangulation facilitated a comprehensive needs assessment and the identification of engagement strategies from different perspectives [17]. External validity might be limited in terms of the sample size and selection of participants—South-Asian Surinamese living in the Dutch city of The Hague—and being mostly first-generation migrants. However, South-Asian Surinamese in the Netherlands mainly live in one of four major cities and the study-design aligns with the study aim. In addition, triangulation of data from the three steps improved the external validity. The support of the key persons—for the recruitment of women, to lead focus group sessions and facilitate interpretation of results—benefited the quality of the study greatly [12]. On the other hand, it could have been an obstacle too—in a selective sample, sensitive issues could remain hidden for the researchers due to cultural norms. The researchers attempted to eliminate this through engaging actively, i.e., as the moderators in the focus groups and through conversations with the key persons as the focus groups progressed [12]. As our key persons were used to the role of being a moderator in discussion meetings within the community, the power imbalance between key persons and focus group participants is regarded as low. The incentive of 15 euro’s for focus group participation to cover travel costs was considered to have no impact on the representativeness of the sample.

### Conclusion

People of South Asian ancestry, such as the South-Asian Surinamese living in the Netherlands, have a disproportionately higher burden of cardiometabolic diseases when compared to other ethnic groups. As South-Asian Surinamese make up one of the largest minority groups in the Netherlands, it is pivotally important to implement culturally tailored preventive interventions for this group. This study sought the preferences and needs of the South-Asian Surinamese, their relatives and health professionals about preventive interventions in a certain area. It has helped to observe a certain mismatch between supply and demand for lifestyle interventions, in particular towards diet and exercise. Incremental changes to current lifestyles; a social network approach and an encouraging approach by professionals seems to be key points for improvement of cardiometabolic prevention.
